# Modular service provision for heterogeneous patient groups: a single case study in chronic Down syndrome care

**DOI:** 10.1186/s12913-019-4545-8

**Published:** 2019-10-21

**Authors:** L. Fransen, V. J. T. Peters, B. R. Meijboom, E. de Vries

**Affiliations:** 1 Department of Tranzo, Tilburg School of Social and Behavioral Sciences, Tilburg University, 5000 LE Tilburg, the Netherlands; 2 Department of Management, Tilburg School of Economics and Management, Tilburg University, 5000 LE Tilburg, the Netherlands; 3 Department of Jeroen Bosch Academy Research, Jeroen Bosch Hospital, PO Box 90153, B1.02.014, 5200 ME ‘s-Hertogenbosch, the Netherlands

**Keywords:** Service modularity, Healthcare, Interfaces, Down syndrome

## Abstract

**Background:**

Service modularity could be promising for organizing healthcare delivery to heterogeneous patient groups because it enables cost reductions while also being responsive towards individual patients’ needs. However, no research on the applicability of modularity in this context exists. To this end, we conducted a qualitative single-case study on chronic healthcare provision for Down syndrome patients, delivered by multidisciplinary pediatric Downteams in the Netherlands, from a modular perspective.

**Methods:**

We conducted six semi-structured interviews with coordinators of multidisciplinary Downteams in six hospitals. In addition, we gathered data by means of observations and analysis of relevant documentation. We transcribed, coded, and analyzed the interviews utilizing the Miles and Huberman approach. The consolidated criteria for reporting qualitative research (COREQ) were applied in this study.

**Results:**

In all six Downteams studied, the modular package for Down syndrome patients (i.e. the visit to the Downteams) could clearly be divided into modules (i.e. the separate consultations with the various professionals), and into different components (i.e. sub-elements of these consultations). These modules and components were linked by different types of customer-flow and information-flow interfaces. These interfaces allowed patients to flow smoothly through the system and allowed for information transfer, respectively.

**Conclusion:**

Our study shows a modular perspective is applicable to analyzing chronic healthcare for a heterogeneous patient group like children with Down syndrome. The decomposition of the various Downteams into modules and components led to mutual insight into each other’s professional practices, both within and across the various Downteams studied. It could be used to increase transparency of delivered care for patients and family. Moreover, it could be used to customize care provision by mixing-and-matching components. More detailed research on chronic modular care provision for patients with DS is needed to explore this.

## Background

Down syndrome (DS) is a complex congenital condition. Individuals with DS share a typical appearance, intellectual disability, and delayed motor development. However, each individual with DS is affected differently by those characteristics. In addition, many individuals with DS experience various DS-related comorbidities. Examples are problems of hearing and vision, autoimmune diseases, airway infections, and heart defects [1]. The prevalence and severity of these comorbidities vary, making patients with DS a very heterogeneous patient group, despite their common genetic background (trisomy 21).

Providing adequate healthcare and interventions in the early life of individuals with DS improves their physical and intellectual abilities [2, 3]. Typically, a multitude of healthcare providers is involved in the care of a child with DS [4]. In the Netherlands, numerous pediatric outpatient clinics organize multidisciplinary team appointments (so-called “Downteams”) for children with DS, including a visit to the pediatrician, speech therapist, physiotherapist and others [4]. These teams differ in their composition and work practices. The extent to which these differences have an influence on healthcare provision is unclear. Besides, various other external healthcare professionals and organizations deliver parts of the required healthcare. This shows the complexity of the care patients with DS have to deal with. Healthcare providers increasingly look for ways to reorganize current DS healthcare provision, while at the same time extending options for adaptation to individual needs.

Modularity promises to relieve problems of complexity in service systems, by its ability to enable efficient customization and responsiveness to individual requirements. Modularity involves the decomposition of a product or service into modules that can be mixed and matched to individual needs, so that each patient receives an *individualized* service package [5, 6]. Modularity has increasingly gained attention in the field of healthcare and studies have been carried out in areas such as elderly care [7], mental care [8] and to a lesser extent hospital care [9]. We addressed the applicability of modularity in chronic DS healthcare provision as an example of complex care in a heterogeneous patient group. We studied whether the dimensions of modularity can be recognized within the service delivery for this type of healthcare, with the potential to make use of modularity theory to meet current demands for reorganization in mind.

### Theoretical background

**Fig. 1:**
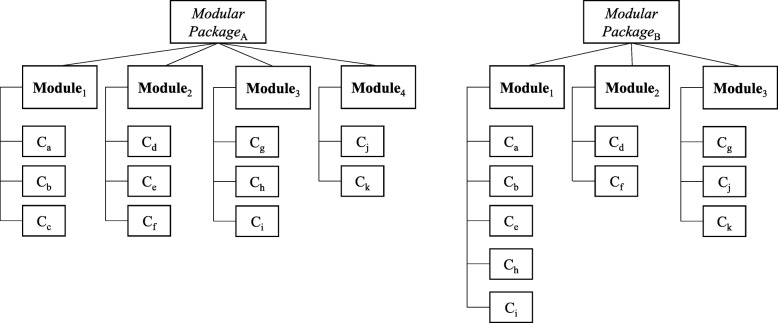
Example of possible modular packages

Modularity originates from the operations management domain. It is a strategy that enables organizations to (re) organize their complex products and services in an efficient way [5]. Modularity concerns the decomposition of these complex products and services into independently functioning modules, each of which consist of separate components. We consider *modules* (M) as separate, relatively independent parts of a service offering with a specific function that can be offered individually, or in combination [10]. Within these modules, standardized *components* (C) can be distinguished, the smallest elements in which a service offering can be meaningfully divided [11]. The mixing-and-matching of various components is referred to as a *modular package* (MP); in healthcare, a modular package is the individualized healthcare package for a patient [7]. Such a modular package can result in an individualized service: the provided modules or components within modules can be adapted to the needs of each individual patient, without necessarily having to change the other parts of the modular package (Fig. 1).

*Interfaces* are important elements of modular services; they provide interaction between modules and between components, how they fit together, and how they connect and interact within the modular package [12, 13]. Interfaces play a major role as linkages in the configuration of modular service provision: they ensure the formation of a functional, coherent whole when *mixing-and-matching* the modules and the components [5]. In services, one can make a distinction between two types of interfaces: information-flow and customer-flow interfaces. *Information-flow* interfaces guide the exchange of information and stimulate information transfer about the (changed) patient situation between the different modules involved in the healthcare provision. *Customer-flow* interfaces enable patients to flow smoothly through the system, which is necessary to provide continuity of care [14]. This is achieved through coordination of activities between providers, and between providers and patients [11].

Interfaces are especially important in service settings. Elements of (healthcare) services are typically consumed at different points in time, and at different locations [15]. Furthermore, modular packages often need reconfiguration, for instance due to patients’ changing healthcare needs. Both the service use at different times and locations, and the potential need for reconfiguration stress the necessity to align the different elements of the service, i.e. the different components and modules. Failing to do so may have serious consequences for quality of care and for the patient’s quality of life [16]. Another characteristic of services is the central role of people: modules and the modular package come into being because of the interaction between service providers (i.e. healthcare professionals) and customers (i.e. patients). The people allow for smooth (re) combinations, thereby acting as interfaces themselves; they play a vital role in healthcare service provision [12].

Modularity is a relatively new concept in the field of healthcare. However, it has great potential because of its possibilities for cost reductions in combination with individualization [17]. Especially the latter is considered important. The possibility to provide healthcare adapted to each individual can potentially contribute to a person-centered approach [18, 19]. This approach, in which healthcare provision is responsive to individual patient preferences, needs, and values, is widely advocated in the Dutch healthcare system [20].

However, healthcare modularity studies are limited and are mostly conducted in the Netherlands and Finland. In the Netherlands, studies focused on elderly care [7, 11, 21, 22] and mental care [8]. In Finland, studies focused on hospital services [9] and the conceptual implications of modularity in health and social services [17, 23]. These studies showed that service modularity may increase customization and efficiency in healthcare, but evidence of these effects in hospital services remains scant. We for the first time applied modularity theory for heterogeneous patient groups in a hospital context by examining whether the dimensions of modularity can be recognized within chronic DS healthcare provision.

## Methods

### Setting of the study

In this paper, we have limited our focus to chronic DS healthcare provision for children in the Netherlands. During childhood, chronic healthcare for individuals with DS is generally coordinated by a pediatrician, preferably as coordinator of a specialized multidisciplinary Downteam [24]. In the Downteam, the pediatrician collaborates with different medical, paramedical and non-medical specialists [25]. These different ‘members’ of the Downteam provide subsequent consultations for children with DS, so that they can visit multiple specialists with knowledge of their condition in 1 day. Healthcare provided by Downteams is generally focused on stimulating the development of the child, physically as well as mentally, and around screening for and coordinating treatment of the various potential comorbidities.

### Study design

We carried out a qualitative, exploratory single case study to test modularity theory in chronic DS healthcare provision for children in the Netherlands. Considering that the topic of study is still in its formative stage, qualitative research in the form of a case study was conducted [26]. Case study research enables one to understand the process, and to answer “how”, “why” and “what” questions [27], which are central in this study. Another advantage of this method is the opportunity to research the study topic in its real-life context [27, 28]; this can contribute to understanding whether a modular approach is feasible in the context of chronic healthcare provision for a heterogeneous patient group.

### Case selection

We took the chronic healthcare provision for children with DS, provided by Downteams, as our case. This type of care serves as an example of chronic care by its wide range of health care professionals and largely heterogeneous patient group. Currently, there are 22 Downteams in the Netherlands [29], located at different hospitals and geographically dispersed over the country. Their set-up and working methods differ from team to team. Best practices for the organization of these teams have not been identified yet; a multidisciplinary guideline with recommendations for the content of the delivered (para) medical care is available, developed under the auspices of the Dutch Pediatric Association [25]; the guideline forms the starting point of healthcare delivery for all Downteams in the Netherlands.

### Data collection

We first collected relevant documents of all 22 Downteams in the Netherlands. We aimed to select a range of Downteams varying in working methods and geographic locations in order to select a representative set of participating Downteams. Based on this aim, the availability of Downteams and by using information from the collected documents, we deliberately selected six out of the 22 Downteams to include in our research. These six Downteams are well-known in the field and provide a good representation of all Downteams in the Netherlands. They were chosen carefully, so that they demonstrated variety in their set-up, working methods and geographic location, leading to a comprehensive view on chronic Down syndrome care. We contacted these six Downteams in writing and by telephone. For these Downteams, we conducted observations and interviews in addition to the documents we collected. The data retrieved from the six Downteams was sufficient for our goal to explore the applicability of modularity principles. The remainder of this section is based on the order in which we retrieved the types of data.

#### Documentation

We collected relevant documentation that was open to the public (e.g. online information brochures on the Downteams, national guideline [25]), and internal documentation of the Downteams (e.g. planning schemes, medical protocols). The collected documents gave valuable information in terms of the set-up and working methods of the Downteam. Hence, documents were assessed first, so that observations and interviews could focus on clarification of the working methods of Downteams and on more detailed topics, such as possibilities for individualization.

#### Observations

In total, two researchers (LF & VP) conducted six observations which lasted half a day, one at each Downteam during consultations of children with DS. We followed a patient at each of his/her (consecutive) consultations (e.g. consultation with pediatrician, consultation with speech therapist). This allowed us to get a better understanding of the care provision. We received oral approval from parents of children with DS and healthcare professionals prior to our observations. The observations were unstructured and focused on the question “*Which dimensions of modularity can be recognized within the service delivery of this Downteam?*”, with the observer as participant. Our aim was to play a neutral role as much as possible. This was appropriate because this type of observation allows the researcher to completely focus on the research and take notes immediately [26]. During those observations, the observer made field notes and theoretical memos that allowed us to summarize the data and collect potential interpretations and relations.

#### Interviews

Interviewees were selected using purposive sampling. We conducted interviews with coordinators (in all cases the pediatrician) from the six selected Downteams. The potential participating coordinators were, prior to the interview, contacted in writing and by telephone. We deliberately chose to interview the coordinators of these Downteams as they had expertise and experience in the field of chronic healthcare provision for children with DS and knowledge on the set-up and working methods of the respective Downteams.

Generally, reaching saturation, meaning new interviews do not yield new data on the interview topics, is considered sufficient for validity [30]. In the case of exploratory studies, a limited amount of interviews can be sufficient [31] in order to get a reliable sense of thematic exhaustion and variability within our data set. In our study, data saturation happened after six interviews, as no new themes emerged from the data gathered between interview five and interview six.

The interviews were semi-structured and lasted approximately 30 min. This was sufficient, as the interviews mostly appeared to confirm the information obtained during document analyses and observations of the Downteams. The semi-structured nature of the interviews allowed us to make sure that important topics were addressed while leaving room for the interviewees to tell their story [26]. In the case that a respondent said something interesting and relevant to our study objective, or the respondent’s answer to our question was not clear, we asked the respondent to clarify the answer. The topic list for the interviews was compiled based on a literature review on (healthcare) modularity and on the collected documentation. See Additional file 1 for the overview of our complete topic list. Because the interviewees were not familiar with the vocabulary of modularity, the wording of questions was adapted to topics relevant to healthcare provision by Downteams. For example, we asked, “*What consultations does the Downteam offer?*” instead of “*Which modules can be distinguished in the Downteam?*” in order to get acknowledged with the specific type of healthcare. In addition, a question like “*To what extent is healthcare provision adapted to the specific patient?*” helped us to check for possibilities of individualization. Interviews were audio-recorded and transcribed.

### Data analysis

The final data consisted of documentation, theoretical memos from the observations and transcripts of the interviews, which we integrated in our analysis. A thematic analysis of the content was carried out using the three steps method described by Miles and Huberman [32]: 1) data reduction; 2) data display, and 3) drawing conclusions/verification. This is a systematic data reduction process building on the reading of transcripts, document summaries and observation notes, segmentation of sentences and phrases, codification of text segments, generation of themes and categories, and identification of relationships [32]. The thematic analysis was guided by our preliminary coding framework and based on definitions derived from modularity literature (See Additional file 2) [33]. Those initial deductive codes were useful in the segmentation and early coding phase of the data analysis. By combining the information from the interviews, observations and document analysis with the theoretical framework, we applied a modular view to the data retrieved. For instance: when the interviewee had mentioned that a consultation with a physiotherapist was also offered independently from the Downteam, we considered this part of the service delivery as a module, as per our definition of modules. The applied framework was continuously discussed and tested during the coding of the interviews [33]. Text segments were compared and contrasted, and codes were assigned. During the analytical phase, all authors of this paper had frequent contact and discussed and assessed the outcomes of the analysis. For example, sometimes it was unclear whether a text segment could be related to the code ‘module’ or the code ‘component’. In order to solve these issues, we used guiding criteria (e.g. what is the respective role of an element in care provision) to determine which text segments belong to a module and belong to a component [34]. Next, data were displayed and compared using data displays (see Results section) that proved useful to see patterns in the collected data. For instance: we collected all the available guidelines for each Downteam in a chart. This helped us to see what is happening and provided opportunities to gain additional in-depth understanding of the data in a convenient way [33]. Based on those displays, a comparison with existing literature was made and conclusions were drawn.

### Quality of the research

To assure internal validity, various measures were taken. First, the concept of modularity and the purpose of the study were extensively explained at the beginning of each interview. As such, interviewees were assisted to provide information relevant towards the goal of the research, increasing the internal validity of the study [26]. Second, the transcripts of the interviews and field notes of the observations were returned to the respondents and we received no comments or corrections. This increased validity because our data was checked by the respondents from whom the data were originally obtained. Third, cross-verification was achieved by using multiple methods (documents, observations and interviews) to analyze the same topic. In that way, different aspects of the topic could be retrieved, hence increasing the validity [27]. Last, reaching data saturation increased the validity of our study [31]. LF and VP determined that this happened after six interviews, because the new interview did not yield new results or adaptations to our coding scheme. With respect to reliability, several measures were taken. First, the COREQ criteria list for qualitative research [35] was used to guide our analysis that was inspired by Miles and Huberman [32]. In doing this, we constantly moved back and forth within the data, the coded extracts of data that we analyzed and the analysis of the data we produced (See Additional file 3 for the complete list). Second, all interviews were conducted in the same manner, by the same person (LF). Moreover, the confidentiality of the information provided by the interviewees was assured at the beginning of the interview. This was done in order to let the interviewee feel at ease and create a comfortable atmosphere. Last, we made use of peer review to assess the quality of our findings [36]: another researcher (VP) analyzed the data independently. The coding results were almost identical, discrepancies were resolved based on the discussion of the researchers. These measures were aimed at decreasing the observer error, and hence increasing the external validity [26].

## Results

During a visit to the Downteam, the patient with DS subsequently meets various (para) medical specialists belonging to the Downteam, within one given part of the day. All Downteams offered a range of consultations from different healthcare professionals to each patient. The teams varied in the duration of the consultations (ranging from 15 to 45 min), the number (ranging from four to eight) as well as the profession of participating healthcare professionals. In three Downteams (A, B & C), all patients visited the same professionals; in the remaining three Downteams (D, E & F) each patient had one or two mandatory consultations with professionals, complemented with consultations with professionals depending on their current needs. Interestingly, the interviewees mentioned that they were not aware of the observed differences between Downteams. They assumed that each Downteam was organized in the same way. Additional file 4 provides a description of the set-up of the participating Downteams.

### Recognizing modularity in the Downteams’ service provision

**Fig. 2:**
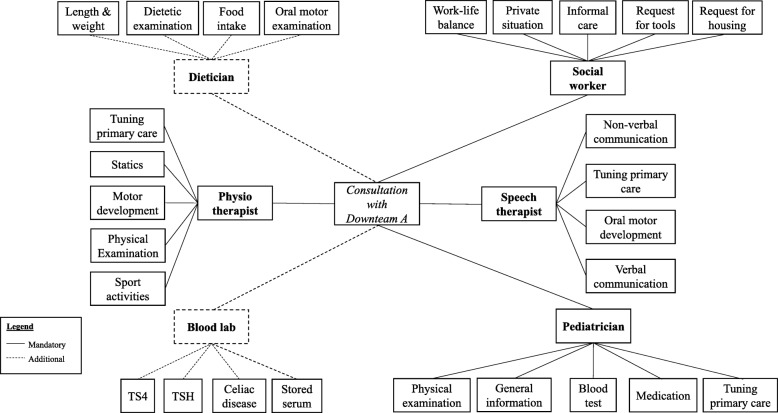
Graphical presentation of a modular package delivered by Downteam A

Based on the information from the documentation, observations and interviews, we were able to describe practices executed by Downteams in modular terms, using the definitions and coding of the text fragments, theoretical memos and documentation as basis. From a modular perspective, the combined consultations of one visit to the Downteam can be seen as the *modular package*, i.e. the healthcare package that each patient with DS visiting the Downteam is provided with. Within this package, the separate consultations with the various healthcare providers form the *modules*. They are indeed independent, as they can also be offered separate from the Downteam. *Components* are elements of the healthcare delivery that have a function on their own but cannot function independently: they are offered as part of the module, in this case, the consultation. The components are based on, for instance, national guidelines set by the Dutch Pediatric Association [25], but can also be based on the Downteam members’ own insights. Examples are ‘physical examination’, ‘oral motor development’, and ‘blood test’. The Downteams studied offered 4 to 8 different modules, wherein various components could be distinguished (a complete picture to illustrate this is shown for Downteam A in Fig. 2; a detailed description of all Downteams is shown in Additional file 4). The content of the components is omitted in Fig. 2, but can be found in Additional file 5.

### Interfaces within Downteams

We found several communication mechanisms that connected the modules (i.e. the different consultations). From a modular perspective, these various ways of communication and connection between the modules can be considered as interfaces. In the Downteams, the planning scheme leading to a convenient order of the modules and the consultation scheme for a particular day are clear examples of customer-flow interfaces. In some Downteams, a letter was sent to patients with DS prior to the visit, asking the parents or relatives of the patient with DS to indicate their preferences with regard to the consultations. By means of this interface, the modular packages were customized to meet the needs of individual patients, guided by professional judgment in the selection of the appropriate modules and components.

**Fig. 3:**
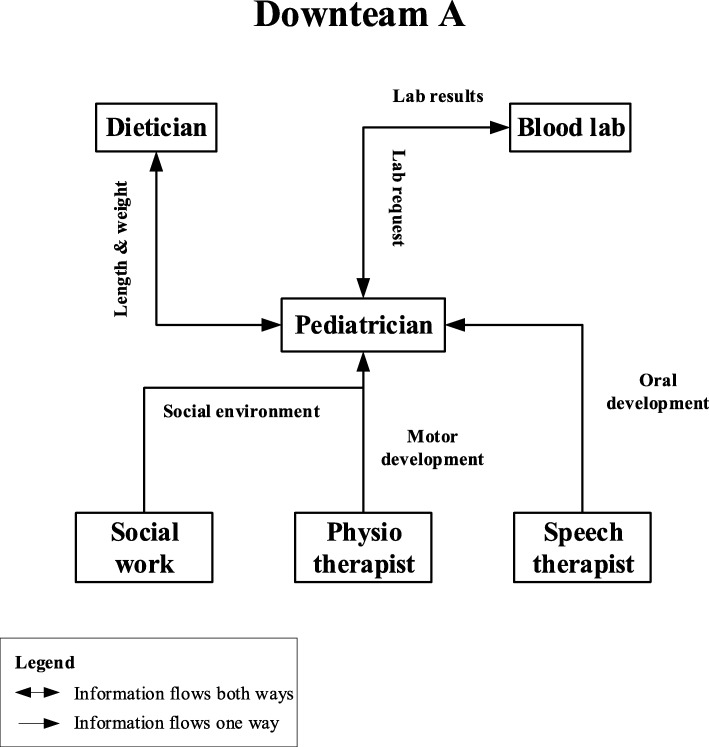
Sample of various communication lines between modules of the modular package of Downteam A

The majority of identified interfaces contributed to information-flow in the modular package. They helped to manage the interaction between the service providers involved in the modular package. Regular multidisciplinary discussion, shared electronic health record (EHR) reports, care plans, direct communication lines and a summarizing letter are clear examples of information-flow interfaces. For example, the shared EHR ensures that the different professionals of the Downteam can read the information reported by other members. The other two interfaces that we observed, a clearly stated work schedule and work protocol, are internal arrangements that allow for predictable interactions between professionals, based on a clear specification of tasks and responsibilities. Figure 3 shows an example of observed communication lines and relevant information that is being shared through these interfaces. The communication lines illustrate the interdependency of the professionals involved in the multidisciplinary setting of DS healthcare provision. For example, the length and weight of the patient is exchanged between the pediatrician and the dietician; it serves as input for general health and growth assessment for the pediatrician, and as input for the analysis of the nutritional status for the dietician.

## Discussion

Our study shows a modular perspective is applicable to analyzing chronic healthcare for a heterogeneous patient group. The modular perspective enabled us to decompose the complex healthcare in the observed Downteams into modules, components and interfaces, and to perform a comparative analysis between these teams, even though they differed considerably. This decomposition creates possibilities to mix-and-match standardized components in order to create individualized modular packages. This implies that every patient can be offered a different combination of components and thus each is treated as unique [7]. In this way, modularity could create a customized service from standardized components. As such, modularity could potentially support person-centered care provision.

Strikingly, the coordinators and other service providers were hardly aware of the different ways in which their and other Downteams are organized. We observed this when we returned our transcripts and results to the interviewees to check for accuracy and resonance with their experiences [37]. Beforehand, they had expected identical results per case, based on the guidelines provided by the Dutch Pediatric Association [25]. The differences we observed were very insightful for them, because the decomposition of the various Downteams into modules and components led to mutual insight into each other’s work practices, both within and across the Downteams we studied. This triggered plans to evaluate and consider restructuring their Downteam, as best practices from other Downteams as well as overlaps and gaps regarding the delivered components within their own team became apparent to every service provider. The modular perspective also increased awareness of the challenges involved in delivering such a complex service: the service providers were not aware of the diversity in interfaces through which the relevant patient information was exchanged.

The modular perspective can also provide transparency to patients and caretakers: it becomes easier to understand the overall healthcare delivery, and where they can best ask their questions. If this perspective is offered to them by means of e.g. a communication map, they can prepare their visit to the Downteam even better.

### Implications for future research

Our exploratory study has some limitations. First, we focused on chronic DS healthcare provision for children in the Netherlands. More studies in other complex care contexts and other countries are needed to assess the external validity of our results.

Second, we only interviewed the coordinators of the Downteams. The remaining involved healthcare professionals (e.g. speech therapist, physiotherapist), patients and their caretakers might have provided additional relevant information into the modular perspective on chronic DS healthcare provision. This could lead to a more comprehensive modular view on this type of complex care. A follow-up study could address this.

Last, we observed a great variety of interfaces in the chronic healthcare provision for patients with DS. This is an important observation to explore further, as a tight fit between the complementary components and the professionals involved will prevent gaps as well as duplications in service provision. This tight fit is achieved through interfaces [13]. Our study has paved the way for more research on this topic, especially on how their dynamics influence care provision.

## Conclusions

In conclusion, we examined whether the dimensions of modularity, a concept from the field of operations management, could be recognized within chronic healthcare service delivery in a heterogeneous patient group (in our case: for children with DS). This was the case: a modular perspective enabled decomposition of the complex healthcare delivered by Downteams into modules and components which could be compared between different Downteams. In this way, this study serves as a first exploration of modularity for a heterogeneous patient group. Future research is needed to assess further potential to individualize care for each patient while also properly linking and aligning interfaces.

## Supplementary information

**Additional file 1.** Topic list.

**Additional file 2.** Coding list.

**Additional file 3.** A 32-item checklist for reporting qualitative studies (COREQ).

**Additional file 4.** Disciplines and consultations in the various Downteams.

**Additional file 5.** Explanation of the modular perspective on Downteam A.

## Data Availability

The datasets generated and analyzed during the current study are not publicly available due to confidentiality but are available from the corresponding author on reasonable request. The data are stored on the secure server of Tilburg University. This server is automatically backed up every 24 h. Backups are stored for 2 weeks.

## Abbreviations

DS: Down syndrome
EHR: Electronic health record
