# Supplementary material for: Modular service provision for heterogeneous patient groups: a single case study in chronic Down syndrome care
Source: BMC Health Serv Res. 2019 Oct 21;19:720. doi: 10.1186/s12913-019-4545-8 (PMC6805608; doi:10.1186/s12913-019-4545-8)
Supplement: Supplementary file 1 — Additional file 1. Topic list. [file 12913_2019_4545_MOESM1_ESM.docx]

Additional file 1. Topic list.

| **Modularity topic** | **Indicative questions** |
| --- | --- |
| Service set-up | What consultations does the Downteam offer? |
|  | How are the consultations organized? |
|  | What standardized practices does each member of the Downteam apply? |
| Service provision | Do all patients visit the same members? If not, how is it determined which patient visits which members? |
|  | Do all patients visit the Downteam with the same frequency? If not, how is it determined how often each patient visits the Downteam? |
| Interfaces in set-up | Is healthcare provision adapted to the specific patient? |
|  | In what ways do the members of the Downteam collaborate? |
|  | Do discussions on ad hoc basis take place? |
|  | Does a multidisciplinary discussion take place, during which all patients are discussed? If so does this discussion take place before, after, or separate from the Downteam? If so, are all members present at this discussion? |
|  | Does the Downteam provide a letter in which all members discuss their findings? If so, is there a standard format used for this letter? |
