# Supplementary material for: Modular service provision for heterogeneous patient groups: a single case study in chronic Down syndrome care
Source: BMC Health Serv Res. 2019 Oct 21;19:720. doi: 10.1186/s12913-019-4545-8 (PMC6805608; doi:10.1186/s12913-019-4545-8)
Supplement: Supplementary file 2 — Additional file 2. Coding list. [file 12913_2019_4545_MOESM2_ESM.docx]

| **Code** | **Sub code** | **Example quotes** |
| --- | --- | --- |
| Standardization | Standardization in care | "The procedures are very standardized", "all patients visit the same members at each visit" "all patients visit the pediatrician and the ophthalmologist, those are standard" "with our team, you can either participate in all consultations, or in none of them" "all consultations have the same duration" "care is very uniform" |
|  | Standardization in frequency visits | "all patients visit the Downteam yearly" |
| Components | - | *All quotes regarding practices within each consultation, e.g.* "I always check the length and weight of the patient" |
| Modules | - | *All quotes regarding the available consultations for patients, e.g.* "Every patient visits the pediatrician, physiotherapist,…, ….,… " "the members of our Downteam are the …. , …., …., " |
| Modular package | - | *All quotes regarding the way that the consultations are combined (i.e. construction of modular package) and all quotes on all consultations that patients (can) have, e.g.* "all patients visit the same professionals: ...., ...., .....," "[our case manager] sends a letter to parents, where parents can […] and they can indicate whom [which professionals] they want to speak to" |
| Interfaces - practices Downteams | O-C interfaces | "[our case manager] sends a letter to parents, where parents can […] and they can indicate whom [which professionals] they want to speak to" |
|  | O-I interfaces | "The EPF gives me information on the consultations with others [members of the Downteam] and, sometimes, I adapt my consultation to that" "We organize a multidisciplinary discussion, at which every member is present .We discuss each patient separately" |
|  | C-C interfaces | "My [pediatricians] consultation is always scheduled after that of the physiotherapist" "At the beginning of the visit, patients are provided with a consultation schedule" |
|  | C-I interfaces | "We adhere to the national guidelines" "the EPF stores all information in one place" "After the multidisciplinary consultation, the secretary forms a letter for which every professional [member of Downteam] writes a part. Next, I [pediatrician] write a conclusion" "Because we are so close to each other, it is easy to just drop by at another member's office and discuss the patient" "the standard format of the letter is that everyone [each member of Downteam] writes their own part" |

Additional file 2. Coding list
