# Supplementary material for: Modular service provision for heterogeneous patient groups: a single case study in chronic Down syndrome care
Source: BMC Health Serv Res. 2019 Oct 21;19:720. doi: 10.1186/s12913-019-4545-8 (PMC6805608; doi:10.1186/s12913-019-4545-8)
Supplement: Supplementary file 3 — Additional file 3. A 32-item checklist for reporting qualitative studies (COREQ). [file 12913_2019_4545_MOESM3_ESM.docx]

Additional file 3. A 32-item checklist for reporting qualitative studies (COREQ).

| **Item** | **Description** | **Check (page number)** |
| --- | --- | --- |
| **Domain 1: Research team and reflexivity** | | |
| *Personal characteristics* | | |
| 1. Interviewer | Which author/s conducted the interviews and/or observations? | Fransen and Peters (p. 18) |
| 2. Credentials | What were the researcher’s credentials? | MSc and MSc (p. 1) |
| 3. Occupation | What was their occupation at the time of the study? | Master student and PhD-student (p. 1) |
| 4. Gender | Was the researcher male or female? | Female and male |
| 5. Experience and training | What experience or training did the researcher have? | Both researchers took a course on Qualitative Research Methods at Tilburg University and had previous experience during thesis writing |
| *Relationship with participants* | | |
| 6. Relationship established | Was a relationship established prior to study commencement? | One of the authors (EV) suggested potential respondents based on her experience in the field of DS healthcare (p. 8) |
| 7. Participant knowledge of the researcher | What did the participants know about the researcher? | The personal interest of the researchers and purpose of the study was explained before the data collection started (p. 12) |
| 8. Researcher characteristics | What characteristics were reported about the researcher? | Interest in research topic, occupation, reason for research (p. 12) |
| **Domain 2: Study design** | | |
| *Theoretical framework* | | |
| 9. Methodological orientation and Theory | What methodological orientation was stated to underpin the study? | Thematic analysis (p. 11) |
| *Participant selection* | | |
| 10. Sampling | How were participants selected? | Purposive sampling (p. 10) |
| 11. Method of approach | How were participants approached? | In writing and by telephone (p. 10) |
| 12. Sample size | How many participants were in the study? | Six (p. 10) |
| 13. Non-participation | How many people refused to participate or dropped out? | 0 |
| *Setting* | | |
| 14. Setting of data collection | Where was the data collected? | Workplace (p. 9) |
| 15. Presence of non-participants | Was anyone else present besides the participants and researchers? | The patient was present during the observations (p. 9) |
| 16. Description of sample | What are the important characteristics of the sample? | Occupation: Pediatrician  (p. 10) |
| *Data collection* | | |
| 17. Interview guide | Were questions, prompts, guides provided by the authors? Was it pilot tested? | The topic list is added to the manuscript as supplementary material (p. 10) |
| 18. Repeat interviews | Were repeat interviews carried out? If yes, how many? | No |
| 19. Audio recording | Did the research use audio recording to collect the data? | Yes (p. 11) |
| 20. Field notes | Were field notes made during and/or after the interview or observation? | Yes (p. 10) |
| 21. Duration | What was the duration of the interviews or observation? | Interviews: 30 minutes (p. 10)  Observation: half-day (p. 9) |
| 22. Data saturation | Was data saturation discussed? | Data saturation was discussed within the research team (p. 10) |
| 23. Transcripts returned | Were transcripts returned to participants for comment and/or correction? | We asked the respondents to review the data we collected. We received no comments and/or corrections (p. 12) |
| **Domain 3: Analysis and findings** | | |
| *Data analysis* | | |
| 24. Number of data coders | How many data coders coded the data? | Two (LF and VP) (p. 13) |
| 25. Description of the coding list | Did authors provide a description of the coding list? | The coding list is added to the manuscript as supplementary material (p. 11) |
| 26. Derivation of themes | Were themes identified in advance or derived from the data? | Themes were identified in advance (p. 11) |
| 27. Software | What software, if applicable, was used to manage the data? | N/A |
| 28. Participant checking | Did participants provide feedback on the findings? | We asked the respondents to review the researchers’ interpretation of the interview data (p. 16) |
| *Reporting* | | |
| 29. Quotations presented | Were participant quotations presented to illustrate the themes/findings? Was each quotation identified? | We made use of examples from the interviews and observations to show the richness of the data (p. 14) |
| 30. Data and findings consistent | Was there consistency between the data presented and the findings? | We present an analytic story where we highlight the key concepts of the study and how our findings shed light on the concepts (p. 13-15) |
| 31. Clarity of major themes | Were major themes clearly presented in the findings? | We used headers in the text to indicate the major themes (p. 13-15) |
| 32. Clarity of minor themes | Is there a description of diverse cases or discussion of minor themes? | We used sub sections in the text to indicate the minor themes (p. 13-15) |
