# Supplementary material for: Modular service provision for heterogeneous patient groups: a single case study in chronic Down syndrome care
Source: BMC Health Serv Res. 2019 Oct 21;19:720. doi: 10.1186/s12913-019-4545-8 (PMC6805608; doi:10.1186/s12913-019-4545-8)
Supplement: Supplementary file 4 — Additional file 4. Disciplines and consultations in the various Downteams. [file 12913_2019_4545_MOESM4_ESM.docx]

Additional file 4. Disciplines and consultations in the various Downteams.

| **Downteam** | **A** | **B** | **C** | **D** | **E** | **F** |
| --- | --- | --- | --- | --- | --- | --- |
| **Mandatory consultation(s)** | Pediatrician, physiotherapist, speech therapist, social worker | Pediatrician, physiotherapist, speech therapist, social worker | Pediatrician, physiotherapist, speech therapist, contact parent, ENT-doctor, ophthalmologist, orthoptist, dietician, social worker, audiologist | Pediatrician, ophthalmologist | Pediatrician | Pediatrician, physiotherapist |
| **Optional consultations** | Dietician, blood lab | N/A | N/A | ENT-doctor, ophthalmologist, orthoptist, physiotherapist, rehabilitation doctor, speech therapist | ENT-doctor, ophthalmologist, orthoptist, physiotherapist, rehabilitation doctor, speech therapist | Speech therapist, special education generalist, dentist, ENT-doctor, ophthalmologist, contact parent |
| **Duration of consultations** | 20 minutes each | 20 minutes each | 20 minutes each | 30 minutes each | 15 - 45 minutes each | 30 minutes each |
