# Supplementary material for: Modular service provision for heterogeneous patient groups: a single case study in chronic Down syndrome care
Source: BMC Health Serv Res. 2019 Oct 21;19:720. doi: 10.1186/s12913-019-4545-8 (PMC6805608; doi:10.1186/s12913-019-4545-8)
Supplement: Supplementary file 5 — Additional file 5. Explanation of the modular perspective on Downteam A. [file 12913_2019_4545_MOESM5_ESM.docx]

Additional file 5. Explanation of the modular perspective on Downteam A.

| **Modular package** | **Module** | **Component** | **Explanation** |
| --- | --- | --- | --- |
| Consultation with Downteam A | Pediatrician | General information  Physical examination  Blood test  Medication  Tuning primary care | Provides the pediatrician with the information necessary to follow the overall growth of a patient  Investigates the body of the patient (e.g. back, feet)  Concerns the discussion on blood samples  Concerns the medication of a patient (e.g. usage, results)  Coordinating and facilitating primary care (e.g. dentist, general practitioner) |
|  | Physio- therapist | Physical examination  Motor development  Statics  Sport  Tuning primary care | Investigates the body of a patient (e.g. back, feet)  Concerns the development of the motion of the patient  Concerns the position and posture of the body of the patient  Concerns issues regarding sporting activities  Coordinating and facilitating with physiotherapist in primary care |
|  | Speech therapist | Oral motor development  Communication (non-verbal)  Communication (verbal)  Tuning primary care | Concerns the oral skills necessary for proper speech and feeding development  Concerns the way the patient communicates in a non-verbal manner  Concerns the way the patient communicates in a verbal manner  Coordinating and facilitating with speech therapist in primary care |
|  | Social worker | Work-life balance  Private situation  Informal care  Requests for tools  Requests for housing | Concerns the balance between work and life issues and whether the family is able to take care of themselves  Concerns any issues regarding the private situation of the patient and his/her family  Concerns the use of informal care by relatives  Concerns any form of procedural issues the patient and his/her family are dealing with regarding tools and housing |
|  | Dietician | Length and weight  Dietetic examination  Food intake  Oral motor examination | Concerns the measurement of length and weight of the patient  Concerns the examination of the patient based on his nutritional problem  Concerns the food intake of the patient  Concerns the oral skills necessary for proper speech and feeding development |
|  | Blood lab | T4  TSH  Celiac disease  Stored serum | Concerns the test for T4  Concerns the test for TSH  Concerns the test for Celiac disease  Concerns the test for Stored serum |
